# Congruence Between Molecular Data and Morphology: Phylogenetic Position of Senodoniini (Coleoptera: Elateridae)

**DOI:** 10.3390/insects10080231

**Published:** 2019-08-01

**Authors:** Robin Kundrata, Alexander S. Prosvirov, Dominik Vondracek, Eliska Sormova

**Affiliations:** 1 Department of Zoology, Faculty of Science, Palacky University, 17. listopadu 50, 771 46 Olomouc, Czech Republic; 2 Department of Entomology, Faculty of Biology, Moscow State University, Leninskie gory 1/12, 119234 Moscow, Russia; 3 Department of Entomology, National Museum, Cirkusová 1740, 193 00 Praha 9—Horní Počernice, Czech Republic

**Keywords:** classification, click beetles, diversity, Lissominae, phylogeny

## Abstract

Senodoniini is a small lineage of click beetles currently comprising 21 species in two genera, distributed in the Himalayas and East and Southeast Asia. The definition and limits of this group have changed considerably during its history. Recent authors treat Senodoniini as a tribe within Dendrometrinae, usually close to Dimini, but this placement has never been rigorously tested. Here, we shed new light on the systematic position and limits of Senodoniini by performing a combined phylogenetic analysis of two nuclear and two mitochondrial molecular markers. Our results recovered Senodoniini not monophyletic, and placed them into the Lissominae complex, where they formed a clade with *Austrelater* Calder & Lawrence (Protelaterini). Molecular phylogeny is in agreement with the adult morphology. Additionally, we examined the morphology of a monotypic genus *Rostricephalus* Fleutiaux from Southeast Asia, which was previously classified in various Elateridae groups including Senodoniini, and its position was always uncertain. This genus shares morphological characters with Protelaterini. We provide morphological redescriptions as well as the figures of main diagnostic characters for *Senodonia* Laporte, *Sossor* Candèze, and *Rostricephalus*. Based on our results, we place these genera to Lissominae: Protelaterini, and hence synonymize Senodoniini Schenkling with Protelaterini Schwarz.

## 1. Introduction

Elateridae are by far the most species-rich family in the beetle superfamily Elateroidea, with approximately 10,000 described species known from all zoogeographical regions [1]. Despite the recent progress in the systematics and phylogenetics of this group, many lineages remain understudied and their phylogenetic placement contentious [2,3,4,5,6]. Senodoniini are a small group with convoluted taxonomic history, including frequently changing limits as well as position in the Elateridae classification [6,7,8]. In most classifications, Senodoniini were placed near Dimini mainly due to the presence of lobate tarsomeres, and the definitions of both groups often overlapped [9,10]. Recently, Senodoniini were considered either a part of the widely delimited Dimini [11], a separate subfamily [8], or a tribe within Dendrometrinae [1,6,12,13].

Senodoniini historically contained several genera from various zoogeographic regions, which are currently placed in various other groups, such as *Penia* Laporte, 1838, *Csikia* Szombathy, 1910, *Neocsikia* Ôhira & Becker, 1972 (all Dendrometrinae: Dimini), *Parallotrius* Candèze, 1878 (Dendrometrinae *incertae sedis*), *Allotriopsis* Champion, 1896 (Elaterinae: Dicrepidiini), *Morostoma* Candèze, 1879 (Morostomatinae), and *Mucromorphus* Ôhira, 1962 (Dendrometrinae: Dendrometrini: Hemicrepidiina) [13]. Currently, this group is restricted to the region encompassing the Himalayas, China, mainland Southeast Asia, and the Sundaland, and contains only *Senodonia* Laporte, 1838 with 20 described species, and a monotypic *Sossor* Candèze, 1883 [8,13]. The enigmatic monotypic genus *Rostricephalus* Fleutiaux, 1918, known also from the East and Southeast Asia, was earlier classified in Senodoniini [14], but then moved to its own subfamily Rostricephalinae [15], Oxynopterinae [16], or Pityobiinae [17], and currently its systematic position remains uncertain [13].

The analyses of a single molecular marker for *Senodonia*, either the 28S rRNA [18,19] or the internal transcribed spacer-2 [20], recovered this genus as a separate lineage not related to Dimini, with an unresolved phylogenetic position. However, Senodoniini were not sampled in the recent morphology-based phylogenetic analyses [3,21] or in the most comprehensive molecular-based analysis of Elateridae to date [5,6]. The aims of this study are (a) to test the monophyly and investigate the phylogenetic position of Senodoniini using the combination of nuclear and mitochondrial molecular markers for *Senodonia* and *Sossor*, (b) to examine in detail the morphology of *Rostricephalus* (for which no DNA-grade material is available) and assess its systematic placement, (c) to re-evaluate the principal diagnostic characters of all above-mentioned genera and update their diagnoses, and (d) to compare the examined genera with closely related taxa revealed by the current phylogenetic analysis.

## 2. Materials and Methods

### 2.1. Taxon Sampling and Laboratory Procedures

We newly sequenced specimens representing both genera currently classified in Senodoniini, that is, *Senodonia* sp. (voucher number UPOL RK1305) and *Sossor hageni* Candèze, 1883 (UPOL RK1304), both from Indonesia, Sumatra. The voucher specimens are deposited in the collection of R. Kundrata, UP Olomouc. Specimens were fixed in 96% alcohol and stored at −20 °C. Whole genomic DNA was extracted using the Genomic DNA Mini Kit (Tissue) (Geneaid Biotech Ltd., New Taipei City, Taiwan) according to the manufacturer protocol but with incubation with GT buffer prolonged to 3 h, incubation with GBT buffer prolonged to 1 h, and the elution performed with 40 µL of elution buffer. Amplifications were performed using PPP Master Mix (Top-Bio, Vestec, Czech Republic). The PCR amplification conditions and list of primers used are given in Table S1. Two nuclear markers, 18S rRNA (1857–1858 bp) and 28S rRNA (628 bp), and two fragments of the mitochondrial genome, *rrnL* (522–523 bp) and *cox1* mtDNA (723 bp), were sequenced. The PCR products were purified using the ethanol precipitation method, and subsequently sequenced in both forward and reverse directions using the commercial service provided by Macrogen, Netherlands. The newly generated sequences were edited using Geneious 7.1.7 (Biomatters Ltd. Auckland, New Zealand; www.geneious.com) and deposited in GenBank with accession numbers MK834161–MK834168.

### 2.2. Dataset Assembling, Alignment, and Phylogenetic Analyses

To test the phylogenetic position of Senodoniini, we merged newly assembled sequences with the complete Elateridae dataset published by Kundrata et al. [6], which consisted of 151 Elateridae representatives, with 30 Phengodidae and Rhagophthalmidae terminals used as outgroups. For the complete list of taxa used in the analysis and other relevant information, see Kundrata et al. [6] (freely available from www.arthropod-systematics.de or upon request from the first author). Sequences were aligned separately using the MAFFT algorithm with default parameters [22] as implemented in Geneious software. Alignments of the length-invariable protein-coding *cox1* sequences were checked by amino acid translation. The best-fit partitioning schemes and partition-specific substitution models were tested using a greedy algorithm in PartitionFinder 1.1.1 [23] under the corrected Akaike information criterion (AICc). Maximum likelihood (ML) analysis was conducted using RAxML 8.1.24 [24] on the CIPRES web server www.phylo.org [25], with default settings and the partitioning scheme as suggested by PartitionFinder. Branch supports were estimated using the Rapid Bootstrap analysis with 1000 replicates [26]. The Bayesian phylogenetic tree was reconstructed through Bayesian inference (BI) of phylogeny using MrBayes 3.2.6 [27] via the CIPRES portal, with partitioning scheme and nucleotide substitution models as defined by PartitionFinder. The Markov chain Monte Carlo search was performed for 4 × 10^7^ generations. Convergence of the Markov chain was assessed in Tracer 1.5 [28] calculating the effective sample size (ESS) for each parameter. Statistical support for specific clades was obtained by calculating the posterior probability of each monophyletic clade after a 20% burn-in.

### 2.3. Morphology

For a detailed morphological examination of adult specimens of Senodoniini, *Rostricephalus*, Lissominae, and Thylacosterninae, material (or in some cases, detailed photographs of the type specimens) from the following collections were examined: Muséum national d’Histoire naturelle, Paris, France (MNHN), Hungarian Natural History Museum, Budapest, Hungary (HNHM), National Museum, Prague, Czech Republic (NMPC), Museo Civico di Storia Naturale, Genova, Italy (MCNG), Zoological Institute, Russian Academy of Sciences, Saint Petersburg, Russia (ZISP), collection of Alexander S. Prosvirov, Moscow State University, Moscow, Russia (PCAP), collection of Robin Kundrata, Olomouc, Czech Republic (PCRK), Naturalis Biodiversity Center, Leiden, The Netherlands (RMNH), Canadian National Collection of Insects, Arachnids, and Nematodes, Agriculture and Agri-Food Canada, Ottawa, Canada (CNCI), Naturkundemuseum, Erfurt, Germany (NKME), collection of Dmitry N. Fedorenko at A.N. Severtsov Institute of Ecology and Evolution, Russian Academy of Sciences, Moscow, Russia (IEME), Senckenberg Deutsches Entomologisches Institut, Müncheberg, Germany (SDEI), Indian Agricultural Research Institute, New Delhi, India (IARI), Natural History Museum, London, The United Kingdom (BMNH), Royal Belgian Institute of Natural Sciences, Brussels, Belgium (RBINS), Naturhistorisches Museum, Basel, Switzerland (NHMB), and Museum für Naturkunde Berlin, Leibniz-Institut für Evolutions- und Biodiversitätsforschung, Berlin, Germany (MFNB). We examined the material representing almost all genera of Lissominae [29], except the South American *Valdivelater* Lawrence & Arias, 2009 and *Tunon* Arias-Bohart, 2013, for which we used information from the recent literature [30,31]. We also examined the specimens of three (out of five) genera of Thylacosterninae (i.e., *Balgus* Fleutiaux, 1920, *Cussolenis* Fleautiaux, 1918, and *Pterotarsus* Gérin-Méneville, 1829). Further, we examined the type specimens of *Rostricephalus vitalisi* Fleutiaux, 1918 (MNHN), *Sossor hageni* (RMNH), and 13 species of *Senodonia* (BMNH, IARI, MCNG, MFNB, MNHN, NHMB, RBINS, SDEI). Most of the examined specimens were mounted on transparent plastic plates, and some specimens were pinned. The genitalia were removed, cleaned, and fixed under the body of the specimen in plastic microvials with glycerol [32]. The material was studied under a Motic SMZ-143-N2GG stereomicroscope and a Micromed 3 trinocular microscope. Photographs of the beetles and part of the photographs of genitalia were taken using a Canon EOS-6D camera with a Canon MP-E 65 mm lens. Most photographs of genitalia were taken from glycerol mounts using a Micromed 3 trinocular microscope with a ToupCam 16 MP video eyepiece. Extended focus technology was used. Body length of the specimens was measured from the apical margin of the frons to the apices of the elytra using a measuring eyepiece of the stereomicroscope. Morphological terminology follows Calder et al. [21], Lawrence & Arias [30], and Costa et al. [1]. The suprageneric classification of Elateridae follows Kundrata et al. [6].

## 3. Results

### 3.1. Alignment Parameters and Phylogenetic Analyses

The aligned dataset of 183 terminals contained 4025 homologous positions (1971, 776, 565, and 723 positions for 18S, 28S, rrnL, and cox1, respectively), including 2594 conserved, 1363 variable, and 1118 parsimony informative characters. PartitionFinder identified partitioning by genes and the cox1 codon positions as the optimal scheme, yielding a total of six partitions, with the nucleotide substitution model GTR+I+G selected for all partitions.

**Figure 1:**
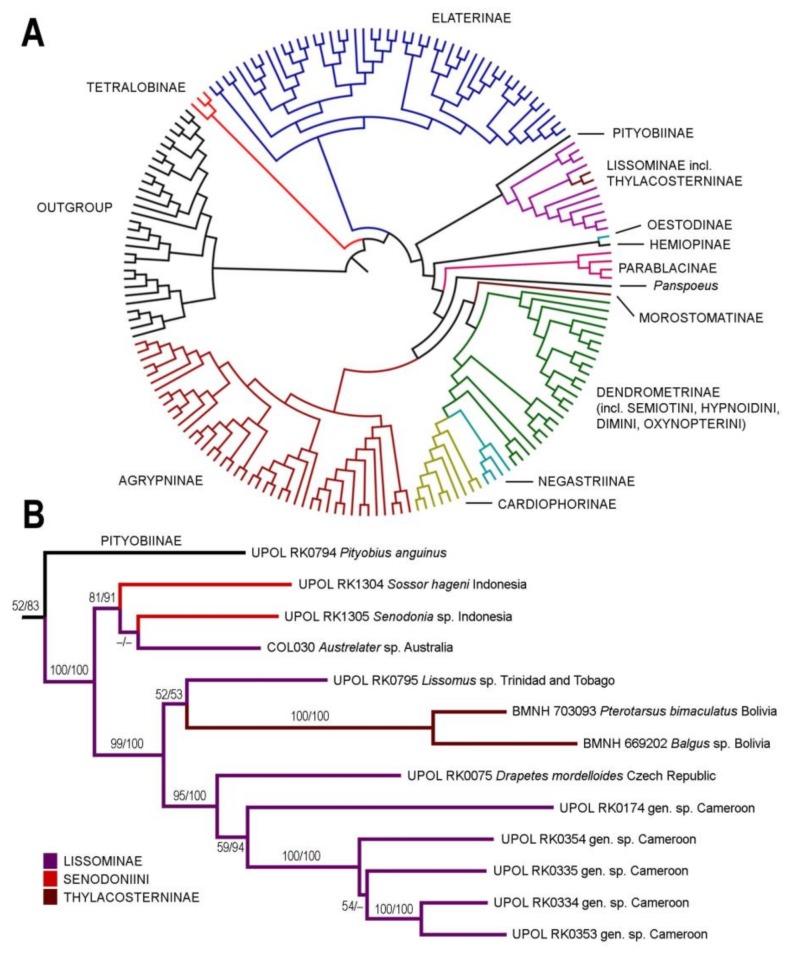
Phylogenetic position of *Senodonia* and *Sossor*. (**A**) Phylogenetic hypothesis for Elateridae based on the 183 terminals aligned by MAFFT and analyzed by maximum likelihood using RAxML. Detailed information on the terminals used is given in Kundrata et al. [6]; (**B**) phylogeny of the Lissominae + Thylacosterninae complex. Numbers at branches represent bootstrap support and Bayesian posterior probabilities, respectively.

Both ML and BI analyses showed identical backbone topology (i.e., clades on a subfamily level and above), only the clade containing Pityobiinae, Senodoniini, Lissominae, and Thyacosterninae was either sister to all Elateridae except Tetralobinae and Elaterinae (ML analysis; Figure 1A) or sister to Elaterinae (Bayesian inference). All families except Dendrometrinae and Lissominae were monophyletic. The ML phylogenetic tree with the highlighted Elateridae subfamilies is given in Figure 1A. In both analyses, Elateridae were monophyletic, and *Senodonia* and *Sossor* were recovered within the maximally supported Lissominae + Thylacosterninae clade. The detail of this clade with statistical support values from both ML and BI analyses is shown in Figure 1B. The first of two subclades of the Lissominae + Thylacosterninae clade contained *Senodonia*, *Sossor*, and *Austrelater* Calder & Lawrence in Calder et al. [21], and obtained a moderate support. The relationships among genera within this subclade remain unclear since each analysis identified a different genus from *Senodonia* and *Sossor* as a sister group to *Austrelater*. The second subclade was strongly supported and contained *Lissomus* Dalman, 1824, *Drapetes* Dejean, 1821, Thylacosterninae, and the unidentified African lissomines (Figure 1B). For details of the remaining clades, we refer to the Supplementary Figure 1 (i.e., the full-resolution ML phylogenetic tree) in Kundrata et al. [6] (freely available from www.arthropod-systematics.de or upon a request from the first author).

### 3.2. Taxonomy

Family Elateridae Leach, 1815
Subfamily Lissominae Laporte, 1835
Tribe Protelaterini Schwarz, 1902
= Senodoniini Schenkling, 1927, **syn. nov.**
= Sphaenelaterini Stibick, 1979

**Figure 2:**
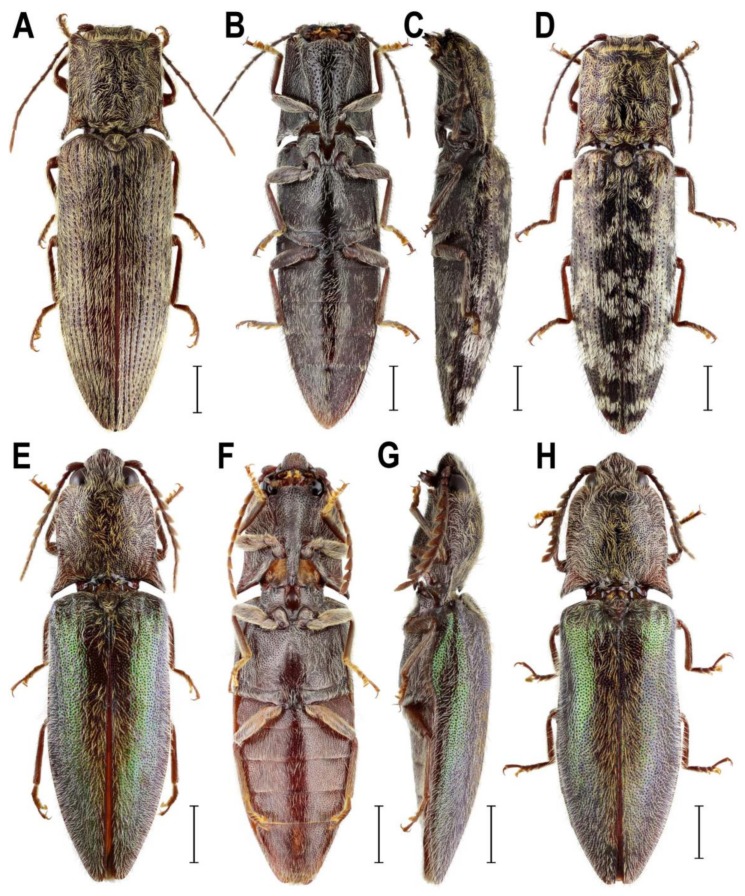
Habitus of *Senodonia* spp. and *Sossor hageni*. *Senodonia jeanvoinei*, Vietnam, male; (**A**) dorsal view. *Senodonia* cf. *quadricollis*, Indonesia, female; (**B**) ventral view; (**C**) lateral view; (**D**) dorsal view. *Sossor hageni*, Indonesia, male; (**E**) dorsal view; (**F**) ventral view; (**G**) lateral view. Female; (**H**) dorsal view. Scale bars = (**A**–**H**): 2 mm.

**Figure 3:**
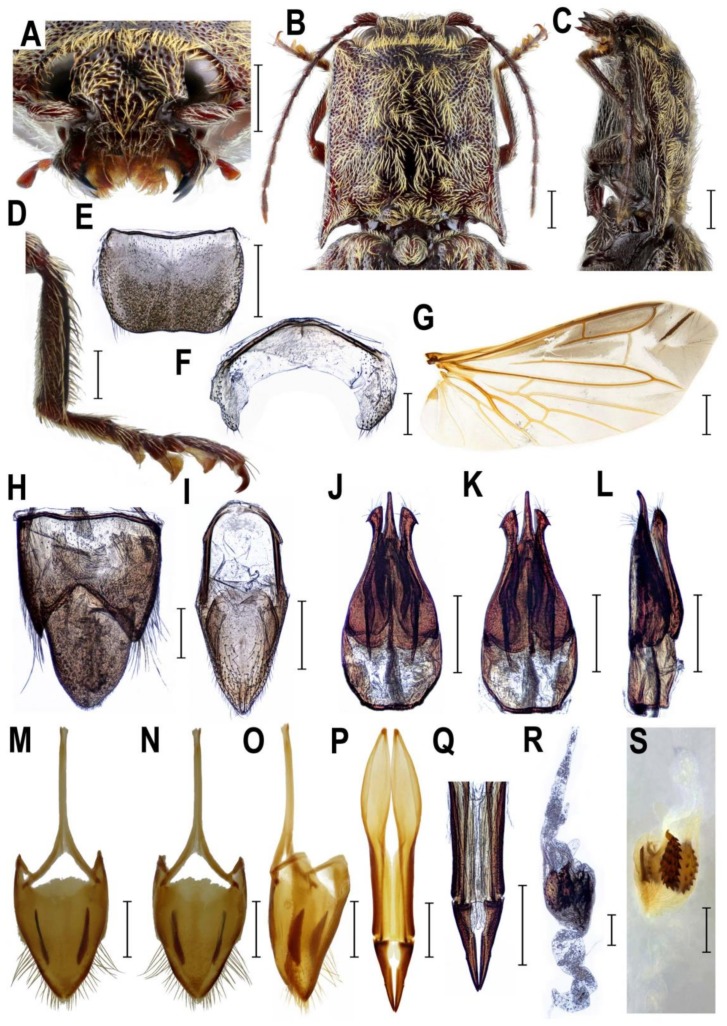
Morphology of *Senodonia* spp. *Senodonia* cf. *quadricollis*, Indonesia, female (**A**–**D**,**G**,**M**–**S**), *Senodonia jeanvoinei*, Vietnam, male (**E**,**F**,**H**–**L**). (**A**) head, frontal view; (**B**) pronotum, dorsal view; (**C**) prosternum, lateral view; (**D**) right metatibia and metatarsus; (**E**) male abdominal tergite VIII; (**F**) male abdominal sternite VIII; (**G**) hind wing; (**H**) male abdominal tergites IX–X; (**I**) male abdominal sternite IX; (**J**) aedeagus, dorsal view; (**K**) aedeagus, ventral view; (**L**) aedeagus, lateral view; (**M**) female abdominal sternite VIII, dorsal view; (**N**) female abdominal sternite VIII, ventral view; (**O**) female abdominal sternite VIII, lateral view; (**P**) ovipositor; (**Q**) apical part of ovipositor; (**R**,**S**) bursa copulatrix. Scale bars = (**G**): 2 mm; (**A**–**C**,**E**,**I**–**S**): 1 mm; (**D**,**F**,**H**): 0.5 mm.

**Figure 4:**
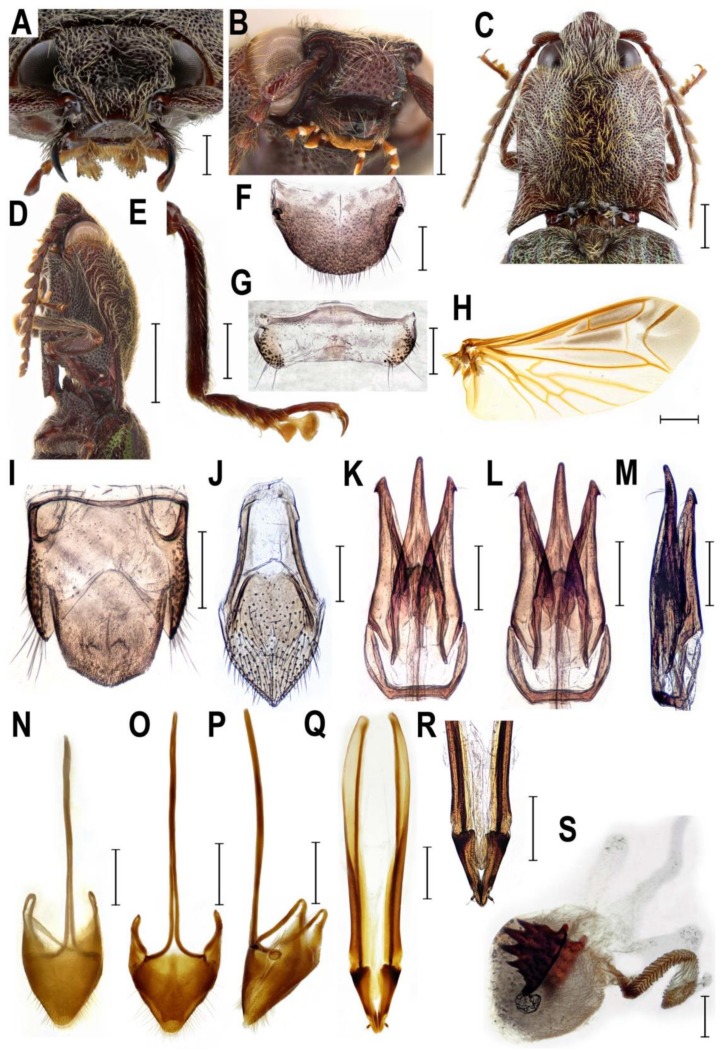
Morphology of *Sossor hageni*, Indonesia, female (**A**,**N**–**S**), male (**B**–**M**). (**A**) head, frontal view; (**B**) head, frontoventral view; (**C**) pronotum, dorsal view; (**D**) prosternum, lateral view; (**E**) right metatibia and metatarsus; (**F**) male abdominal tergite VIII; (**G**) male abdominal sternite VIII; (**H**) hind wing; (**I**) male abdominal tergites IX–X; (**J**) male abdominal sternite IX; (**K**) aedeagus, dorsal view; (**L**) aedeagus, ventral view; (**M**) aedeagus, lateral view; (**N**) female abdominal sternite VIII, dorsal view; (**O**) female abdominal sternite VIII, ventral view; (**P**) female abdominal sternite VIII, lateral view; (**Q**) ovipositor; (**R**) apical part of ovipositor; (**S**) bursa copulatrix. Scale bars = (**D**,**H**): 2 mm; (**C**,**N**–**R**): 1 mm; (**A**–**B**,**E**–**G**,**I**–**M**); S: 0.5 mm.

**Figure 5:**
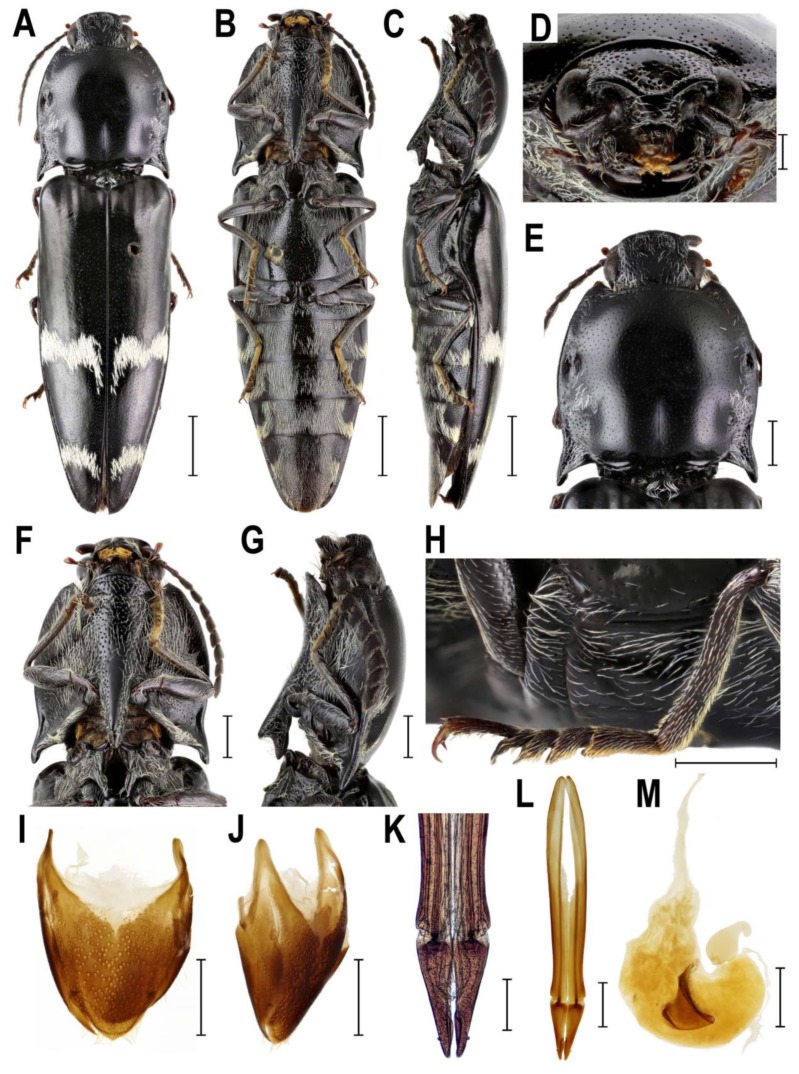
Morphology of *Rostricephalus vitalisi*, Vietnam, female. (**A**) habitus, dorsal view; (**B**) habitus, ventral view; (**C**) habitus, lateral view; (**D**) head, frontoventral view; (**E**) pronotum, dorsal view; (**F**) prosternum, ventral view; (**G**) prosternum, lateral view; (**H**) right metatibia and metatarsus; (**I**) abdominal sternite VIII, dorsal view; (**J**) abdominal sternite VIII, lateral view; (**K**) apical part of ovipositor; (**L**) ovipositor; (**M**) bursa copulatrix. Scale bars = (**A**–**C**): 2 mm; (**E**–**J**,**L**–**M**): 1 mm; (**D**,**K**): 0.5 mm.

**Figure 6:**
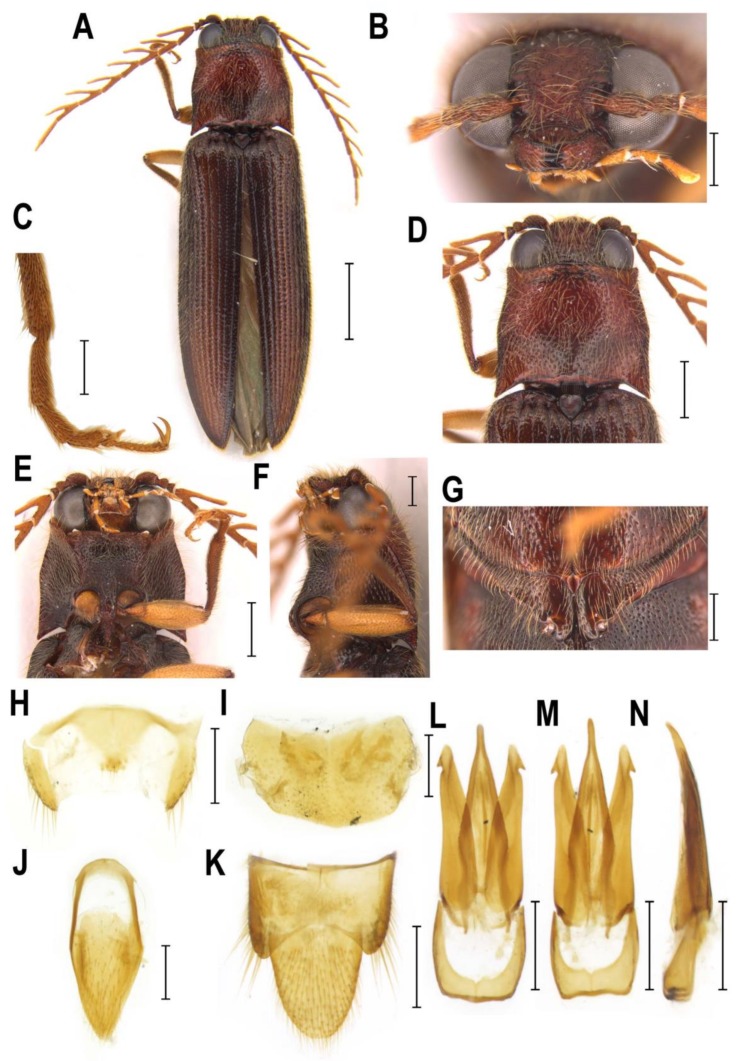
Morphology of *Austrelater* sp., Australia, male. (**A**) habitus, dorsal view; (**B**) head, frontal view; (**C**) right metatibia and metatarsus; (**D**) pronotum, dorsal view; (**E**) prosternum, ventral view; (**F**) prosternum, lateral view; (**G**) metacoxal plate; (**H**) abdominal sternite VIII; (**I**) abdominal tergite VIII; (**J**) abdominal sternite IX; (**K**) abdominal tergites IX–X; (**L**) aedeagus, dorsal view; (**M**) aedeagus, ventral view; (**N**) aedeagus, lateral view. Scale bars = (**A**): 2 mm; (**D**–**E**): 1 mm; (**B**–**C**,**F**–**N**): 0.5 mm.

**Figure 7:**
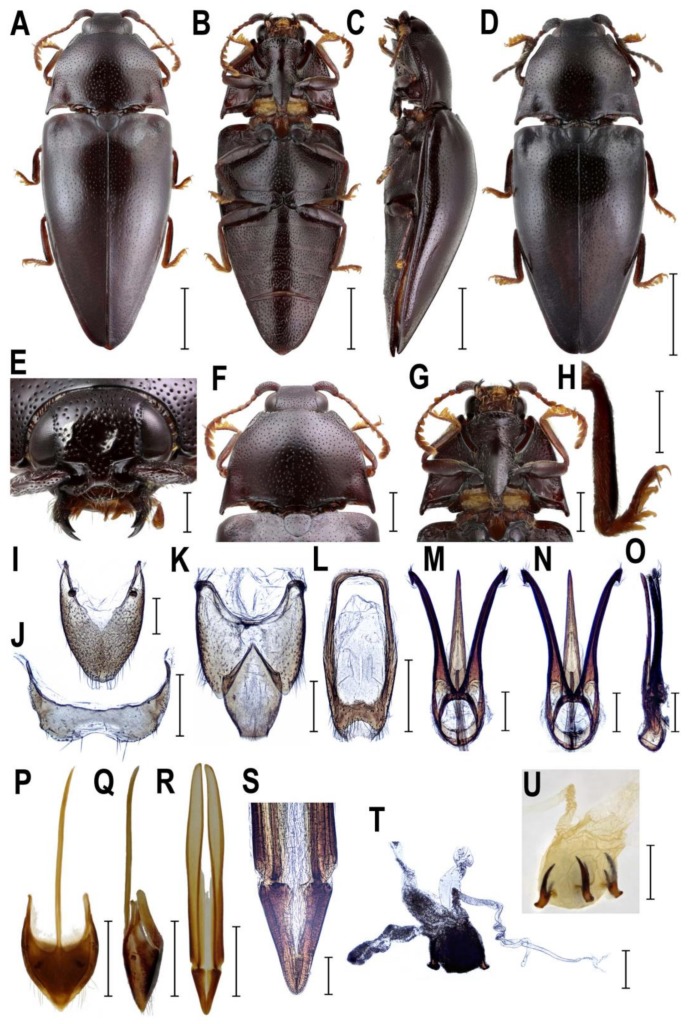
Morphology of *Lissomus* sp., Peru, male (**A**–**C**,**E**–**O**), female (**D**,**P**–**U**). (**A**) habitus, dorsal view; (**B**) habitus, ventral view; (**C**) habitus, lateral view; (**D**) habitus, dorsal view; (**E**) head, frontal view; (**F**) pronotum, dorsal view; (**G**) prosternum, ventral view; (**H**) right metatibia and metatarsus; (**I**) male abdominal tergite VIII; (**J**) male abdominal sternite VIII; (**K**) male abdominal tergites IX–X; (**L**) male abdominal sternite IX; (**M**) aedeagus, dorsal view; (**N**) aedeagus, ventral view; (**O**) aedeagus, lateral view; (**P**) female abdominal sternite VIII, dorsal view; (**Q**) female abdominal sternite VIII, lateral view; (**R**) ovipositor; (**S**) apical part of ovipositor; (**T**, **U**) bursa copulatrix. Scale bars = (**A**–**D**): 2 mm; (**F**–**G**,**L**,**P**–**R**,**T**–**U**): 1 mm; (**E**,**H**–**K**,**M**–**O**): 0.5 mm; (**S**): 0.2 mm.

**Remark 1**. Based on the results of molecular phylogenetic analysis and the detailed examination of morphological characters, genera *Senodonia* Laporte, 1838, *Sossor* Candèze, 1883 (both Senodoniini), and *Rostricephalus* Fleutiaux, 1918 (Oxynopterinae; based on morphology alone) are here transferred to Lissominae: Protelaterini, and Senodoniini Schenkling 1927 is considered a subjective junior synonym of Protelaterini Schwarz, 1902. For further details, see Discussion. For the comparison of principal diagnostic characters of *Senodonia*, *Sossor*, *Rostricephalus*, *Austrelater* (Protelaterini; forms a clade with *Senodonia* and *Sossor*; Figure 1), and *Lissomus* (type genus of Lissominae), see Figure 2, Figure 3, Figure 4, Figure 5, Figure 6 and Figure 7.

#### 3.2.1. Genus *Senodonia* Laporte, 1838

*Senodonia* Laporte, 1838: 12. Type species: *Senodonia quadricollis* Laporte, 1838, by monotypy.
=*Allotrius* Laporte, 1840: 231. Type species: *Allotrius quadricollis* Laporte, 1838, by monotypy.
=*Hemiolimerus* Candèze, 1863: 227. Type species: *Hemiolimerus emodi* Candèze, 1863, by monotypy.
=*Orientis* Vats & Kashyap, 1992: 252. Type species: *Orientis montanus* Vats & Kashyap, 1992, by monotypy.

**Diagnostic redescription**. **Adult** (Figure 2A–D and Figure 3A–S). Body 11–22 mm long, elongate, subparallel-sided, moderately convex, pale to dark brown, clothed with rather dense long setae often forming patches on pronotum and elytra (Figure 2A–D). Mouthparts directed antero-ventrally (Figure 3A). Mandibular apex broad when viewed anteriorly, perpendicular to plane of movement. Frons slightly or moderately broadly concave medially. Frontoclypeal region more or less flat, not produced forwards between antennal insertions (Figure 3A). Labrum fully exposed. Antenna serrate from antennomere IV in male; antennomere I more or less straight (Figure 3B), inserted into shallow saucer-shaped depression; dorsal rim not completely carinate from eye to base of labrum (Figure 3A). Antennal insertions medium-sized, separated by approximately 1.5–2.5 diameters. Hypomeron without pockets for reception of antennae (Figure 2B). Pronotosternal sutures almost straight. Prosternum produced forwards to form pronounced rounded chin piece; chin piece curved and separate by impression from rest of prosternum. Prosternal process slightly incurved behind procoxae, with small subapical tooth; rather long behind procoxae (Figure 2B and Figure 3C). Mesoventrite more or less elongate, procoxal rests moderately developed, mesoventral process relatively short and wide, apically almost straight. Mesocoxal cavity open to both mesanepisternum and mesepimeron; mesepimeron weakly transverse. Mesocoxae rather widely separated. Mesotrochanter rather short (Figure 2B). Elytral epipleura narrow at basal one-third; elytra punctate-striate. Apex of hind wing with single oblique sclerotization (Figure 3G). Metacoxal plates more or less narrow, weakly narrowed outwards. Tibia subcylindrical, not compressed laterally. Tarsomeres I–IV each with distinct lamella ventrally (Figure 3D). Disc of tergite X in male covered by distinct microtrichia only, without setae (Figure 3H). Aedeagal median lobe notably broadened at basal part, unevenly narrowed to apex (Figure 3J–L). Sternite VIII and tergite VIII in female fused basally by thin strip of sclerotization, usually with two longitudinal inner sclerites situated between tergite VIII and sternite VIII (Figure 3M–O). Ovipositor rather stout; paraprocts more or less broadened, moderately elongate; gonocoxites strongly sclerotized, with small styli attached subapically or medially (Figure 3P,Q). Bursa copulatrix with at least two pairs of spiny plate-like sclerites (Figure 3R,S). **Immature stages**. For the brief description and drawings of larval head and last abdominal segments of *S. sculpticollis* (Fairmaire, 1888), see Dolin [33].

**Species included**. Twenty described and several undescribed species; for more information, see the annotated catalogue by Kundrata et al. [13].

**Distribution**. Bhutan, Cambodia, China, India, Indonesia, Laos, Malaysia, Myanmar, Nepal, Thailand, Vietnam [13].

#### 3.2.2. Genus *Sossor* Candèze, 1883

*Sossor* Candèze, 1883: 208. Type species: *Sossor hageni* Candèze, 1883, by monotypy.

**Diagnostic redescription**. **Adult** (Figure 2E–H and Figure 4A–S). Body 15–19 mm long, elongate, subparallel-sided, moderately convex, chestnut brown, with greenish elytra, clothed with rather dense long setae (Figure 2E–H). Mouthparts directed ventrally (Figure 4A,B). Mandibular apex broad when viewed anteriorly, perpendicular to plane of movement. Frons broadly and deeply concave medially. Frontoclypeal region strongly produced forwards between antennal insertions, then declivous backwards to base of labrum (Figure 4A,B). Labrum partially covered by frontoclypeal region. Antenna serrate from antennomere IV in male; antennomere I more or less straight (Figure 4C), inserted into deep saucer-shaped depression; dorsal rim not completely carinate from eye to base of labrum (Figure 4A,B). Antennal insertions medium-sized, separated by approximately 2.0–2.5 diameters. Hypomeron without pockets for reception of antennae (Figure 2F). Pronotosternal sutures almost straight. Prosternum produced forwards to form pronounced rounded chin piece; chin piece curved and separate by impression from rest of prosternum. Prosternal process slightly incurved behind procoxae, with small subapical tooth; rather long behind procoxae (Figure 2F and Figure 4D). Mesoventrite more or less elongate, procoxal rests moderately developed, mesoventral process relatively short and wide, apically almost straight. Mesocoxal cavity open to both mesanepisternum and mesepimeron; mesepimeron weakly transverse. Mesocoxae rather narrowly separated. Mesotrochanter rather short (Figure 2F). Elytral epipleura narrow at basal one-third; elytra without defined striae. Apex of hind wing with a single crescent-shaped sclerotization (Figure 4H). Metacoxal plates more or less narrow, almost parallel-sided. Tibia subcylindrical, not compressed laterally. Tarsomeres I–IV each with distinct lamella ventrally (Figure 4E). Disc of tergite X in male covered by distinct microtrichia only, without setae (Figure 4I). Aedeagal median lobe weakly broadened at basal part, gradually narrowed to apex (Figure 4K–M). Sternite VIII and tergite VIII in female fused basally by thin strip of sclerotization (Figure 4N–P). Ovipositor rather stout; paraprocts more or less broadened, moderately elongate; gonocoxites strongly sclerotized, with small subapical styli (Figure 4Q,R). Bursa copulatrix with paired spiny plate-like sclerites (Figure 4S). **Immature stages**. Unknown.

**Species included**. Monotypic genus, with only *S. hageni*.

**Distribution**. Indonesia (Sumatra), Malaysia (Sabah) [9,13].

#### 3.2.3. Genus *Rostricephalus* Fleutiaux, 1918

*Rostricephalus* Fleutiaux, 1918: 252. Type species: *Rostricephalus vitalisi* Fleutiaux, 1918, by monotypy.

**Diagnostic redescription**. **Adult** (Figure 5A–M). Body 15–17 mm long, elongate, subparallel-sided, moderately convex, blackish, partially clothed with rather long whitish setae forming two transversal bands behind middle of each elytron (Figure 5A–C). Mouthparts directed antero-ventrally (Figure 5D). Mandibular apex broad when viewed anteriorly, perpendicular to plane of movement. Frons broadly and deeply concave medially. Frontoclypeal region moderately produced forwards between antennal insertions, then declivous to base of labrum (Figure 5D). Labrum partially covered by frontoclypeal region. Antenna serrate from antennomere IV in male; antennomere I more or less straight, inserted into very deep saucer-shaped depression; dorsal rim not completely carinate from eye to base of labrum (Figure 5D). Antennal insertions very large, separated by less than one diameter. Hypomeron without pockets for reception of antennae (Figure 5B,F,G). Pronotosternal sutures almost straight. Prosternum produced forwards to form pronounced rounded chin piece; chin piece curved and separate by impression from rest of prosternum. Prosternal process slightly incurved behind procoxae, with large subapical tooth; rather long behind procoxae (Figure 5F,G). Mesoventrite more or less elongate, procoxal rests moderately developed, mesoventral process relatively short and wide, apically almost straight. Mesocoxal cavity open to both mesanepisternum and mesepimeron; mesepimeron weakly transverse. Mesocoxae rather widely separated. Mesotrochanter moderately elongate (Figure 5B). Elytral epipleura broad at basal one-third; elytra without defined striae. Metacoxal plates more or less narrow, almost parallel-sided. Tibia subcylindrical, not compressed laterally. Tarsomeres I–IV each with distinct lamella ventrally (Figure 5H). Aedeagal median lobe weakly broadened at basal part, gradually narrowed to apex [34]. Sternite VIII and tergite VIII fused basally by thin strip of sclerotization (Figure 5I,J). Ovipositor rather stout; paraprocts more or less broadened, moderately elongate; gonocoxites strongly sclerotized, with small subapical styli (Figure 5K,L). Bursa copulatrix with paired plate-like sclerites (Figure 5M). **Immature stages**. Unknown.

**Species included**. Monotypic genus, with only *R. vitalisi*.

**Distribution**. Cambodia, China (Taiwan), Laos, Vietnam [34,35,36].

## 4. Discussion

### 4.1. Systematic Position and Morphology of Senodoniini

Recent studies showed that the molecular analyses may help to understand the phylogenetic relationships of the taxonomically problematic groups in Elateridae [4,5,6,37,38]. Here we used for the first time the combination of nuclear and mitochondrial markers to infer the phylogenetic position of the currently defined Senodoniini (i.e., *Senodonia* and *Sossor*). Our results clearly indicate that these genera are not closely related to Dendrometrinae or to Dimini (Figure 1), which was previously hypothesized by numerous authors [1,9,12,39]. This is supported also by their different morphology; *Senodonia* and *Sossor* have antenna inserted into a saucer-shaped depression (antenna inserted in a small crescent-shaped socket in Dimini), pronotum without a pronounced sublateral carina (pronotum with a complete sublateral carina in Dimini), prosternal process rather long behind procoxae (prosternal process usually short behind procoxae in Dimini), metacoxal plates weakly narrowed outwards or almost parallel-sided (metacoxal plates strongly narrowed outwards in Dimini), tarsomeres I–IV each with a distinct ventral lobe (usually only tarsomeres III and IV lobate ventrally in Dimini), female sternite VIII and tergite VIII basally fused (female sternite VIII and tergite VIII free, connected by membranes in Dimini), ovipositor rather stout, paraprocts more or less broadened and moderately elongate, and gonocoxites strongly sclerotized, with small subapical or medial styli (ovipositor rather long, paraprocts not broadened, strongly elongate, and gonocoxites weakly sclerotized, with pronounced styli attached almost apically in Dimini) (Figure 2B,F, Figure 3A,C,D,O–Q and Figure 4A,B,D,E,P–R) [9,10].

Instead, molecular phylogenetic analysis placed both *Sossor* and *Senodonia* with a maximal statistical support into the Lissominae + Thylacosterninae clade (Figure 1). Jiang et al. [18] and Meng et al. [19] already suggested that Senodoniini are not phylogenetically close to Dimini based on the analyses of the 28S ribosomal rRNA sequences, but their datasets were taxonomically limited and did not include Lissominae and Thylacosterninae. The position of Senodoniini within Lissominae is supported also by their adult morphology. Both *Senodonia* and *Sossor* share with the lissomines mandibular apex broad when viewed anteriorly (perpendicular to plane of movement; also in Thylacosterninae), prosternum produced forwards to form a pronounced rounded chin piece, prosternal process slightly incurved behind procoxae, with a subapical tooth, metacoxal plates more or less narrow, weakly narrowed outwards or almost parallel-sided, tarsomeres I–IV each usually with a distinct lamella (also in Thylacosterninae), sternite VIII and tergite VIII basally fused, ovipositor rather stout, paraprocts usually broadened, and the gonocoxites strongly sclerotized (also in Thylacosterninae) (Figure 2B,F, Figure 3C,D,O–Q, Figure 4D,E,P–R, Figure 6E–G and Figure 7B,C,G,H,Q–S). Additional support for the relationships of *Senodonia* and *Sossor* with Lissominae can be found in the hind wing venation [21,40] (Figure 2G and Figure 3H). Lawrence & Arias [30] already called for the closer examination of Senodoniini since the larvae from Southeast Asia similar to *Senodonia* [33] share with Lissominae several characters including the specially modified spine-like setae on the abdomen. Lissominae are supported only by two larval characters, that is, a complex larval sensorium, which consists of a single subdivided dome, and the patches of modified tergal setae [21,30]. Additionally, all known lissomine larvae share mandibular cutting edge with a bifurcate retinaculum, however, this character is present also in unrelated Oestodinae [21]. Unfortunately, illustrations and the description of *Senodonia* larva by Dolin [33] are not detailed enough to confirm the presence of the above-mentioned characters in this genus, and therefore the more detailed examination of Senodoniini larvae is needed to better understand the immature morphology of the Lissominae complex.

### 4.2. Monophyly, Phylogeny, and Classification of Lissominae

The monophyly of and interrelationships within Lissominae and its tribes are still somewhat contentious, and this group is not supported by any adult synapomorphy [21,33]. Lissominae currently include two tribes: the worldwide Lissomini (*Lissomus*, *Drapetes,* and three smaller genera) and the Protelaterini with a Gondwanan distribution (*Protelater* Sharp, 1877 and *Sphaenelater* Schwarz, 1902 from New Zealand, *Austrelater* from Australia, and three South American genera) [1,12,29,31,33]. Oestodinae, which were often classified together with lissomines [1,16,30], form a separate subfamily not closely related to Lissominae [5,6,29]. On the other hand, the relationships between Lissominae and Thylacosterninae are not well understood [21,41] and in some studies, Thylacosterninae are embedded deeply in Lissominae ([5] this study; Figure 1). What is more, current molecular phylogeny recovered the Senodoniini genera in a relatively well-supported clade with *Austrelater*, together sister to remaining Lissominae and Thylacosterninae (Figure 1B).

Our results are in agreement with the previous morphology-based and molecular phylogenetic analyses which recovered *Austrelater* sister to remaining Lissominae or Lissominae + Thylacosterninae [5,21,41]. Unfortunately, *Austrelater* is the only non-Lissomini representative in the molecular dataset, and more Protelaterini including the type genus need to be sampled to help us understand the limits of the currently defined lissomine tribes.

The adult morphology of *Senodonia* and *Sossor* supports their placement near *Austrelater* in Protelaterini. Therefore, these two genera are transferred here to Protelaterini, with which they share the following characters: antennomere I more or less straight and only moderately long, hypomeron without pockets for reception of antennae, mesocoxal cavity open to both mesanepisternum and mesepimeron, both mesanepisternum and mesepimeron only weakly transverse, mesocoxa more or less elongate, tibia subcylindrical and not compressed laterally, and stylus small, palpiform or button-shaped, attached subapically or medially (Figure 2, Figure 3, Figure 4 and Figure 6). Molecular phylogeny suggests *Senodonia* and *Sossor* are probably not a monophyletic group (Figure 1), and this is supported by a number of morphological characters in which they differ. For example, *Senodonia* have mouthparts directed antero-ventrally (mouthparts directed ventrally in *Sossor*), frons slightly or moderately broadly concave medially (frons deeply concave medially in *Sossor*), frontoclypeal region more or less flat, not produced forwards between antennal insertions (frontoclypeal region strongly produced forwards in *Sossor*), labrum fully exposed (labrum partially covered by frontoclypeal region in *Sossor*), antenna inserted into a shallow saucer-shaped depression (antenna inserted into a deep saucer-shaped depression in *Sossor*), elytra punctate-striate (elytra without defined striae in *Sossor*), metacoxal plates weakly narrowed outwards (metacoxal plates almost parallel-sided in *Sossor*), aedeagal median lobe distinctly broadened at basal part and unevenly narrowed to apex (aedeagal median lobe slightly broadened at basal part and gradually narrowed to apex in *Sossor*), abdominal segment VIII in female usually with two longitudinal inner sclerites (segment VIII in female without these sclerites in *Sossor*), and the bursa copulatrix with at least two pairs of spiny plate-like sclerites (bursa copulatrix with only one pair of spiny plate-like sclerites in *Sossor*).

However, since the relationships between *Senodonia*, *Sossor,* and *Austrelater* are not fully resolved and statistically supported, we postpone the discussion on the Senodoniini monophyly and their exact position in Lissominae until the detailed analysis including more material and using the wider range of markers is available. To elucidate the relationships within the Lissominae + Thylacosterninae complex, and to provide a natural classification of the group, it is crucial to sequence Protelaterini from New Zealand and South America, including the type genus *Protelater*.

### 4.3. Systematic Position and Morphology of Rostricephalus

The placement of *Rostricephalus* in the Elateridae classification has always been problematic and this enigmatic genus was at various times included in several subfamilies like Senodoniinae, Rostricephalinae, Oxynopterinae, and Pityobiinae [1,12,14,15,16,17,35,39]. Kundrata et al. [5] redefined Pityobiinae without *Rostricephalus*, and so this genus remained tentatively in Oxynopterinae, although no comprehensive morphological study was carried out, and it differs significantly from Oxynopterinae in a number of characters like, for example, the shape of frontoclypeal region, mouthparts, antennal insertions, pronotum, metacoxal plates, tarsomeres, female pregenital segments, and genitalia.

Interestingly, in the original description, Fleutiaux [42] placed *Rostricephalus* close to *Protelater*, which is currently classified in Lissominae: Protelaterini [29,30]. The detailed morphological investigation of *Rostricephalus* supports the close relationships of this genus with Lissominae including Senodoniini. Characters supporting the inclusion of *Rostricephalus* into Lissominae include the mandibular apex broad when viewed anteriorly (perpendicular to plane of movement), metacoxal plates more or less narrow, almost parallel-sided, tarsomeres I–IV each with a distinct lamella ventrally, sternite VIII and tergite VIII in female basally fused, ovipositor rather stout, with paraprocts more or less broadened and only moderately elongate, and the gonocoxites strongly sclerotized (Figure 5; see Figure 2, Figure 3, Figure 4, Figure 6 and Figure 7 for other Lissominae). Within the Lissominae, *Rostricephalus* is morphologically most similar to some Protelaterini, especially *Anaspasis* Candèze, 1881 and *Protelater*. These genera share with *Rostricephalus* the frontoclypeal region moderately produced forwards between antennae and partially covering labrum, the antenna inserted into a deep saucer-shaped depression, with a dorsal rim at least partially carinate from eye to base of labrum, the antennal insertions large, separated by one diameter or less, the prosternal sutures almost straight, the mesotrochanter moderately elongate, aedeagal median lobe not broadened at basal part and gradually narrowed to apex, and bursa copulatrix with paired sclerites.

Based on the above-mentioned morphological evidence, here we transfer *Rostricephalus* from Oxynopterinae to Lissominae: Protelaterini. Finding of the DNA-grade material as well as the larva of *Rostricephalus* could help to elucidate the detailed phylogenetic placement of this genus within Lissominae.

## 5. Conclusions

The first multigene phylogenetic analysis of Senodoniini clearly indicates that this group is not closely related to Dendrometrinae: Dimini, which were regularly suggested as its nearest relatives. Instead, both *Senodonia* and *Sossor* were embedded with a maximal statistical support in the Lissominae + Thylacosterninae clade. This placement is additionally supported by the morphological characters of adults, and probably also of larvae [30]. The monophyly of the currently defined Senodoniini is questioned by our data. Both *Senodonia* and *Sossor*, along with the yet unsequenced *Rostricephalus*, share diagnostic morphological characters with Protelaterini. Therefore, we place all three above-mentioned genera to Lissominae: Protelaterini, synonymizing Senodoniini with Protelaterini under Lissominae.

## Supplementary Materials

The following are available online at https://www.mdpi.com/2075-4450/10/8/231/s1, Table S1: Primers used for PCR amplification of the studied gene fragments.
