# Supplementary material for: Congruence Between Molecular Data and Morphology: Phylogenetic Position of Senodoniini (Coleoptera: Elateridae)
Source: Insects. 2019 Aug 1;10(8):231. doi: 10.3390/insects10080231 (PMC6723317; doi:10.3390/insects10080231)
Supplement: Supplementary file 1 [file insects-10-00231-s001.pdf]

Table S1. Primers used for PCR amplification* of the studied gene fragments.

Gene	Code	Sequence (5' >> 3')
18S rRNA	fragment A	
	18S 5'	GACAACCTGGTTGATCCTGCCAGT
	18S b5.0	TAACCGCAACAACTTTAAT
	fragment B	
	18S ai	CCTGAGAAACGGCTACCACATC
	18S b2.5.1	CGTTTTTGGCAAATGCTTTCGC
	fragment C	
	18S a1.0.1	GGTGAAATTCTTGGATCGTC
	18S bi	GAGTCTCGTTCGTTATCGGA
	fragment D	
	18S a2.0	ATGGTTGCAAAGCTGAAAC
	18S 3'Irk1	TACGACTTTTACTTCCTCTA
28S rRNA	28S ff	TTACACACTCCTTAGCGGAT
	28S dd	GGGACCCGTCTTGAAACAC
rrnL mtDNA	16Sar	CGCCTGTTTAACAAAAACAT
	16SB2	CTCCGGTTTGAACTCAGATCA
cox1-3' mtDNA	S-Jerry	CAACATYTATTYTGATTYTTTGG
	S-Pat	GCACTAWTCTGCCATATTAGA

* PCR amplification conditions:

18S rRNA: initialization at 94°C for 2 minutes, followed by 40 cycles as follows: denaturation at 94°C for 30 seconds, annealing at 45°C for 30 seconds, elongation at 72°C for 105 seconds, and final elongation at 72°C for 10 minutes;

28S rRNA: initialization at 94°C for 2 minutes, followed by 40 cycles as follows: denaturation at 94°C for 30 seconds, annealing at 50°C for 45 seconds, elongation at 72°C for 105 seconds, and final elongation at 72°C for 10 minutes;

rrnL mtDNA: initialization at 94°C for 3 minutes, followed by 34 cycles as follows: denaturation at 94°C for 30 seconds, annealing at 51°C for 45 seconds, elongation at 72°C for 60 seconds, and final elongation at 72°C for 8 minutes;

cox1 mtDNA: initialization at 95°C for 5 minutes, followed by 40 cycles as follows: denaturation at 95°C for 30 seconds, annealing at 50°C for 45 seconds, elongation at 72°C for 2 minutes, and final elongation at 72°C for 10 minutes.
